# Immune mechanisms in chronic kidney disease-mineral and bone disorder: current insights and therapeutic implications

**DOI:** 10.3389/fmed.2025.1678640

**Published:** 2025-10-09

**Authors:** Bin Xu, Rui Ma, Yuqiang Wu, Chi Liu, Xiangrong Song

**Affiliations:** ^1^Department of Orthopedics, Deyang Fifth Hospital, Deyang, Sichuan, China; ^2^Graduate School of Xinjiang Medical University, Ürümqi, Xinjiang, China; ^3^Department of Nephrology and Institute of Nephrology, Sichuan Provincial People’s Hospital, Sichuan Clinical Research Centre for Kidney Diseases, Chengdu, China; ^4^Department of Nephrology, Deyang Sixth People’s Hospital, Deyang, Sichuan, China

**Keywords:** chronic kidney disease-mineral and bone disorder, immune dysregulation, vascular calcification, osteoimmunology, fibroblast growth factor 23

## Abstract

Chronic kidney disease-mineral and bone disorder (CKD-MBD) is recognized as a systemic syndrome that manifests with a range of complications including mineral dysregulation, skeletal abnormalities, and vascular calcification (VC). Recent research has increasingly pointed toward immune dysregulation as a pivotal factor in the development and progression of this disorder. The current review endeavors to consolidate the latest findings regarding how chronic inflammation, dysfunction of immune cells, and disturbances in the gut-kidney axis contribute to the progression of CKD-MBD. Central to the mechanisms at play are pro-inflammatory cytokines, such as tumor necrosis factor-α (TNF-α) and interleukin (IL)-6, which are found to facilitate bone resorption through the activation of the receptor activator of NF-kappaB ligand (RANKL)/receptor activator of nuclear factor-kappa B (RANK)/osteoprotegerin (OPG) signaling pathway. Furthermore, macrophage-induced VC is linked to the activation of the NLR family pyrin domain containing 3 (NLRP3) inflammasome. Additionally, an imbalance between osteoblasts and osteoclasts, driven by uremic toxins, exacerbates the skeletal manifestations of the disorder. Despite the availability of current therapeutic options, including phosphate binders and vitamin D analogs, these treatments fall short in adequately addressing the immune-mediated aspects of CKD-MBD, indicating an urgent need for innovative strategies that effectively target inflammatory pathways, inhibit sclerostin, or modulate fibroblast growth factor (FGF)-23 levels. Emerging preclinical studies have shown that sodium-glucose cotransporter 2 (SGLT2) inhibitors and anti-sclerostin antibodies hold significant promise in lessening VC and enhancing bone health. However, translating these findings into clinical application encounters hurdles related to the diversity of patient populations and the dependence on surrogate endpoints for efficacy. This review emphasizes the critical need for incorporating immune-centric strategies into the management of CKD-MBD. It advocates for the development of biomarker-driven, personalized therapies and highlights the importance of conducting longitudinal studies to bridge the existing gaps in knowledge and improve patient outcomes.

## 1 Introduction

Chronic kidney disease (CKD) represents a progressive disorder marked by ongoing kidney damage or a diminished glomerular filtration rate that persists for more than 3 months (1). It is frequently indicated by the presence of albuminuria or structural abnormalities in the kidneys. CKD poses a significant global health concern, impacting more than 10% of the global population, with its prevalence continuing to rise due to aging demographics and increasing risk factors such as diabetes and hypertension (2). In the Asian region, approximately 434.3 million adults are affected by CKD, with China and India together representing 69.1% of the region’s total cases, indicating notable regional disparities (3). The global impact of this disease disproportionately burdens lower socioeconomic groups, who face higher prevalence rates, limited access to medical care, and poorer health outcomes, thus highlighting CKD as a critical equity issue (4). The increasing prevalence of this epidemic, coupled with its links to cardiovascular morbidity and mortality, stresses the urgent requirement for further exploration of its underlying mechanisms, such as chronic kidney disease-mineral and bone disorder (CKD-MBD), to guide prevention and management approaches.

According to the Kidney Disease Improving Global Outcomes (KDIGO) guidelines, CKD-MBD is a systemic syndrome characterized by abnormalities in bone histology and ectopic calcification. This condition fundamentally arises from an imbalance in mineral metabolism parameters, including calcium, phosphorus, parathyroid hormone (PTH), vitamin D, and fibroblast growth factor-23 (FGF-23). Such imbalances impede the bone remodeling process and promote vascular calcification (VC), which significantly impacts patients with stage 5 CKD (5–7). This condition is almost universally observed in patients with advanced CKD, leading to increased risks of fractures, cardiovascular incidents, and higher mortality rates (8). The importance of CKD-MBD stems from its function as a crucial factor in poor health outcomes, with VC–arising from phosphate retention and inflammation–being a major contributor to cardiovascular death among CKD patients (9). Additionally, beyond the susceptibility to bone fractures, CKD-MBD indicates a wider systemic issue, aggravated by new elements such as inflammation and alterations in gut microbiota, which further challenge its treatment. Prompt detection and focused treatment approaches are essential, as CKD-MBD not only deteriorates quality of life but also highlights the pressing need for novel therapeutic methods to alleviate its significant clinical ramifications.

Chronic kidney disease-mineral and bone disorder exhibits a complex interplay with the immune system, driven by chronic inflammation and gut dysbiosis, which significantly influence mineral metabolism and cardiovascular outcomes in CKD patients. Recent evidence underscores that CKD triggers persistent immune activation through uremic toxins, oxidative stress (OS), and microbial imbalances, fostering an inflammatory environment that exacerbates bone resorption and VC, hallmark features of CKD-MBD (5). Pro-inflammatory cytokines, such as Interleukin (IL)-1 and tumor necrosis factor-α (TNF-α), are pivotal in promoting these pathological processes, linking immune dysregulation to skeletal and vascular complications (10). Furthermore, gut dysbiosis compromises intestinal barrier integrity, increasing bacterial product translocation, which amplifies systemic inflammation and perpetuates CKD-MBD progression (5). FGF-23, a critical regulator of mineral metabolism, also interacts with immune cells, modulating inflammatory responses and highlighting a bidirectional relationship between CKD-MBD and immunity (11). This intricate connection suggests that immune-mediated pathways, including inflammation and osteoimmunological mechanisms, are central to CKD-MBD pathogenesis, offering potential therapeutic targets to mitigate its impact. These findings emphasize the need for integrated approaches addressing both immune and mineral dysregulation to improve outcomes in CKD.

This review examines advances in understanding the immune mechanisms of CKD-MBD and explores emerging therapies to mitigate its clinical burden. This review explores the advancements in understanding the immune mechanisms underlying CKD-MBD and investigates emerging therapies aimed at alleviating its clinical burden. It synthesizes the latest evidence from PubMed/Medline, Web of Science, and Cochrane Library databases up to January 2025, focusing on the immune mechanisms, inflammation, and therapeutic strategies related to CKD-MBD.

## 2 Pathophysiology of CKD-MBD

### 2.1 Mineral metabolism dysregulation

CKD disrupts systemic mineral homeostasis, primarily through impaired phosphate excretion and reduced renal synthesis of active vitamin D. Hyperphosphatemia, a hallmark of CKD-MBD, triggers FGF-23 elevation to promote urinary phosphate excretion, but progressive renal failure limits compensatory mechanisms, exacerbating phosphate retention (12). CKD also impacts the parathyroid’s internal circadian clock, contributing to the hyperplasia of the parathyroid gland in these patients (13). The calcium-sensing receptor (CaSR), found within the parathyroid gland, acts as the primary regulator of PTH release (14). Notably, recent findings indicate that the CaSR possesses a phosphate-binding site; when phosphate binds to this site, it modifies the receptor’s configuration, driving it into an inactive state and consequently initiating PTH secretion (15). Small fluctuations in plasma ionized calcium are promptly adjusted through the movement of calcium on the surface of bones. In contrast, plasma phosphate levels tend to vary more significantly and respond more slowly to conditions of hyperphosphatemia (16). Concurrently, reduced renal 1α-hydroxylase activity causes vitamin D deficiency, impairing intestinal calcium absorption and contributing to hypocalcemia (17). Secondary hyperparathyroidism (sHPT) develops as hypocalcemia and vitamin D deficiency stimulate PTH secretion, further worsening bone and vascular pathology (5). Emerging evidence also highlights the role of uremic toxins such as indoxyl sulfate (IS) in suppressing klotho expression, amplifying FGF-23 resistance and perpetuating mineral dysregulation (18). These interconnected disturbances create a vicious cycle, driving CKD-MBD progression (19).

### 2.2 Skeletal abnormalities

Chronic kidney disease-mineral and bone disorder induces heterogeneous bone disorders, ranging from high-turnover osteitis fibrosa to low-turnover adynamic bone disease (ABD). Early CKD stages often exhibit ABD, characterized by suppressed osteoblast activity due to uremic toxin-mediated inhibition of Wnt/β-catenin signaling and elevated sclerostin levels (20). As CKD progresses, sustained sHPT may override these inhibitory signals, leading to excessive bone resorption and osteitis fibrosa (21). Osteomalacia, marked by defective mineralization, is linked to vitamin D deficiency and elevated FGF-23, which impair phosphate availability for bone matrix formation (22). Notably, FGF-23 directly suppresses osteocyte differentiation, exacerbating bone fragility (22). These abnormalities collectively increase fracture risk and correlate with poor clinical outcomes, including cardiovascular mortality (23). In CKD-MBD, the primary clinical features of osteomalacia are associated with defects in bone mineralization. This is particularly evident in tumor-induced osteomalacia (TIO), which arises from hypophosphatemia and FGF-23-related mechanisms, resulting in dysfunction and limited activity (24, 25). High-turnover bone disease is a prevalent form of renal osteodystrophy, characterized by accelerated bone formation and resorption. Elevated PTH levels are indicative of this condition and correlate with bone density and fracture risk (26). ABD represents the most common form of renal osteodystrophy; although asymptomatic, it is closely linked to a poor prognosis, including an increased fracture risk. Fractures can occur independently of VC or premature death, and treatment with anti-resorptive agents may not effectively mitigate this risk. Furthermore, a low bone turnover state can be diagnosed using markers such as the sclerostin/iPTH ratio, which correlates with impaired bone mass and quality (27). The core characteristics of osteoporosis involve the deterioration of bone quality and density, which are directly related to mineral metabolism disorders, such as elevated PTH levels and decreased bone material quality (21, 28, 29).

### 2.3 Vascular calcification

Vascular calcification, a hallmark of CKD-MBD, arises from the osteogenic transformation of vascular smooth muscle cells (VSMCs) driven by hyperphosphatemia, inflammation, and uremic toxins. Elevated phosphate activates RUNX2 in VSMCs, promoting calcium-phosphate deposition and arterial stiffening (23). Pro-inflammatory cytokines (e.g., TNF-α) and OS further enhance VC by upregulating bone morphogenetic proteins (BMPs) and downregulating calcification inhibitors like matrix Gla protein (17). Uremic toxins, such as IS, exacerbate VSMC apoptosis and extracellular vesicle release, accelerating microcalcification (5). Clinically, VC is strongly associated with cardiovascular mortality, as calcified vessels impair hemodynamics and increase cardiac afterload (30). Emerging biomarkers, including miR-125b-2-3p and sulfatase 1 (SULF1), show promise in predicting VC severity, highlighting opportunities for early intervention (23).

**FIGURE 1:**
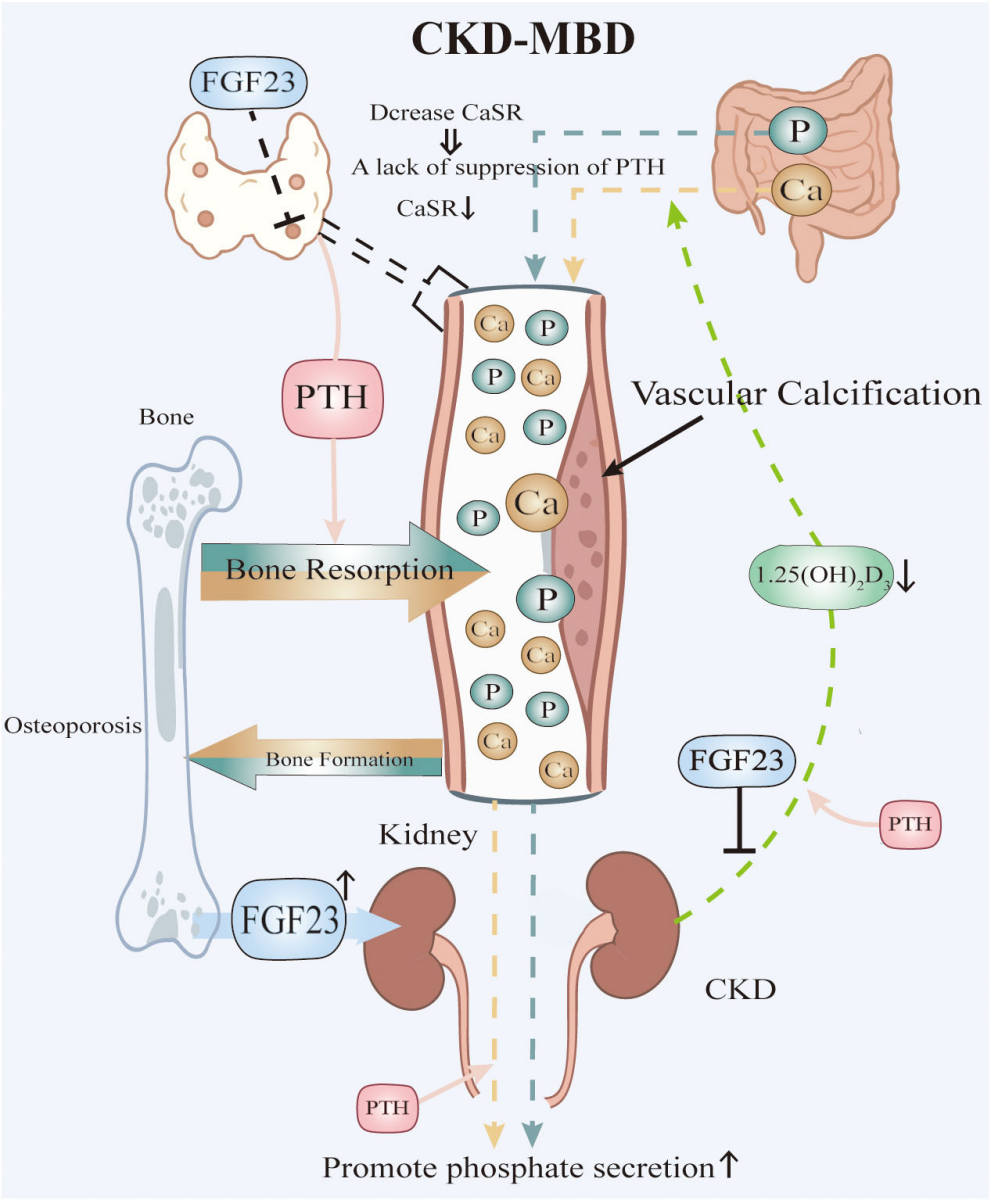
Pathophysiological Interplay in CKD-MBD. Elevated phosphate levels, resulting from renal failure, stimulate the secretion of FGF23. High levels of FGF23 suppress the synthesis of active vitamin D and contribute to the downregulation of CaSR in the parathyroid glands, thereby impairing calcium sensing. The combination of low active vitamin D and impaired calcium sensing leads to increased secretion of PTH, resulting in sHPT. Both PTH and FGF23 promote bone resorption, which releases additional calcium and phosphate into the bloodstream. However, renal impairment hinders adequate phosphate excretion, despite elevated levels of FGF23 and PTH, thereby perpetuating hyperphosphatemia. Additionally, low levels of active vitamin D reduce intestinal calcium absorption. The resultant mineral imbalances, characterized by hyperphosphatemia and fluctuations between hypercalcemia and hypocalcemia, together with inflammation and other contributing factors, drive vascular calcification and disrupt normal bone remodeling.

Figure 1 delineates the dysregulated mineral metabolism axis in CKD, wherein FGF-23 induces parathyroid hyperplasia and downregulates calcium-sensing receptors (CaSR), abrogating calcium-mediated suppression of PTH secretion (15); consequent PTH excess stimulates bone resorption but fails to promote phosphaturia due to renal impairment, while FGF-23 exacerbates hyperphosphatemia through dual mechanisms (11, 14): suppressing renal 1α-hydroxylase activity to reduce calcitriol (1,25-dihydroxyvitamin D3) synthesis and directly enhancing renal phosphate excretion, thereby establishing a vicious cycle of calcitriol deficiency that further aggravates secondary hyperparathyroidism through loss of vitamin D receptor (VDR)-mediated transcriptional repression and impairs intestinal calcium absorption, collectively driving hypercalcemia, hyperphosphatemia, progressive VC, and uncoupled bone remodeling characterized by increased resorption and suppressed formation, with therapeutic calcitriol supplementation representing a targeted intervention to partially correct this axis.

## 3 CKD-associated immune dysregulation

### 3.1 Chronic inflammation and oxidative stress

Chronic kidney disease is marked by a persistent state of low-grade inflammation, a critical factor driving disease progression and its associated complications, such as cardiovascular disease (CVD) (31). This chronic inflammatory state is largely fueled by elevated levels of pro-inflammatory cytokines, notably IL-6 and TNF-α. IL-6, a central mediator of the acute-phase response, is significantly increased in CKD and correlates strongly with disease severity. It amplifies systemic inflammation by triggering the production of acute-phase proteins and is implicated in heightened cardiovascular risk, a leading cause of mortality in CKD patients (32). Similarly, TNF-α exacerbates renal injury by promoting apoptosis of renal tubular cells and stimulating the release of additional inflammatory mediators, thus perpetuating a pro-inflammatory environment (33). These cytokines not only reflect the inflammatory burden but also actively contribute to tissue damage and fibrosis, key hallmarks of CKD progression.

Oxidative stress, another defining feature of CKD, significantly amplifies this inflammatory milieu and impairs immune function. The accumulation of reactive oxygen species (ROS) in CKD results from an imbalance between oxidant production and antioxidant defenses, damaging cellular components such as lipids, proteins, and DNA. This oxidative burden activates inflammatory pathways, including the nuclear factor-kappa B (NF-κB) system, which further upregulate cytokine production, creating a vicious cycle of inflammation and oxidative damage (34). Moreover, OS disrupts immune homeostasis, leading to a dysregulated immune response characterized by both hyperactivation and immunosuppression. For instance, ROS-mediated damage impairs the function of immune cells, reducing their ability to effectively combat infections while simultaneously promoting chronic inflammation (5). This dual impact underscores the complex interplay between OS and inflammation, accelerating renal damage and contributing to the high morbidity observed in CKD. Understanding these mechanisms is pivotal for devising strategies to mitigate inflammation and its deleterious effects in this population.

### 3.2 Immune cell dysfunction and the gut-kidney axis in CKD

**FIGURE 2:**
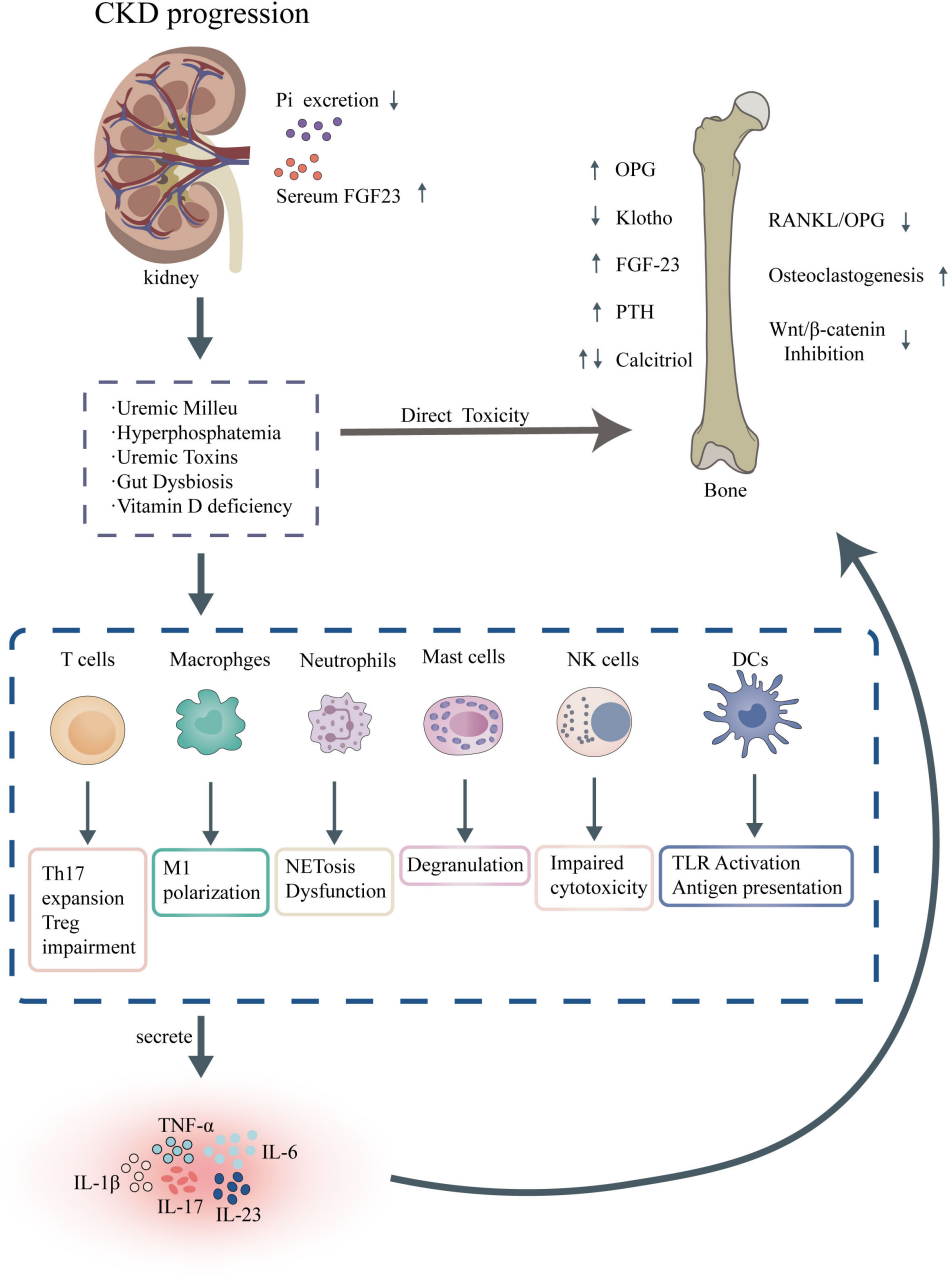
Pathophysiological mechanisms of CKD-MBD and associated immune dysregulation.

Immune cell dysfunction is a key driver of CKD progression (Figure 2), involving abnormalities in adaptive and innate immune cells, including T cells, B cells, and monocytes/macrophages. T cell populations in CKD exhibit a shift toward pro-inflammatory phenotypes, with reduced regulatory T cell function, which fails to suppress excessive immune responses, thereby sustaining inflammation and tissue injury (5). B cell dysregulation further compounds this immune imbalance, characterized by altered antibody production and increased autoantibody formation, which may exacerbate renal damage through immune complex deposition (35). Monocytes and macrophages, critical components of the innate immune system, show heightened activation and polarization toward pro-inflammatory M1 phenotypes in CKD. This shift promotes the production of fibrogenic cytokines, such as transforming growth factor-beta (TGF-β), driving renal fibrosis and tubular injury (36). These cellular abnormalities collectively contribute to a maladaptive immune environment that accelerates CKD progression.

Dysbiosis of the gut microbiota leads to the disruption of tryptophan metabolism (resulting in the production of toxins such as IS and the entry of lipopolysaccharides (LPS) into the bloodstream (37). This process activates the AhR/TLR4 complex, which in turn triggers the activation of the NF-κB pathway, resulting in the release of pro-inflammatory factors and the generation of ROS, along with mitochondrial damage (38, 39). Subsequently, the NLRP3 inflammasome is assembled, leading to the activation of caspase-1 (40). This cascade results in the release of IL-1β and IL-18, pyroptosis, VC, and disturbances in bone metabolism, ultimately contributing to the progression of CKD-MBD (41).

## 4 Immune mechanisms in CKD-MBD

Chronic kidney disease-mineral and bone disorder is a systemic condition marked by disruptions in mineral metabolism, bone pathology, and VC, intricately linked to immune dysregulation. The interplay between immune responses and CKD-MBD pathogenesis has gained significant attention, particularly in how inflammation drives bone resorption and VC. This section delves into the immune mechanisms underpinning CKD-MBD, focusing on inflammatory pathways promoting bone resorption, immune-mediated VC, and the effects of uremic toxins on bone and immune cells, synthesizing recent findings to elucidate their roles in disease progression.

### 4.1 Inflammatory pathways promoting bone resorption

#### 4.1.1 The RANKL/RANK/OPG axis and inflammatory pathways

The receptor activator of nuclear factor-κB ligand (RANKL)/receptor activator of nuclear factor-Kb (RANK)/osteoprotegerin (OPG) axis is a cornerstone of bone remodeling, and its dysregulation in CKD-MBD significantly contributes to bone resorption. RANKL, expressed by osteoblasts and activated T cells, binds to RANK on osteoclast precursors, stimulating their differentiation into mature osteoclasts and enhancing bone resorption (42). In CKD, systemic inflammation elevates pro-inflammatory cytokines such as IL-6 and TNF-α, which upregulate RANKL expression while suppressing OPG, a decoy receptor that curbs RANKL activity, thereby tipping the balance toward osteoclastogenesis (43). PTH, frequently elevated in CKD, further amplifies this process by boosting RANKL production, exacerbating bone loss. Recent studies in young rats with experimental CKD demonstrate that serum PTH levels correlate with increased RANKL and decreased OPG, alongside altered bone geometry and strength, underscoring the axis’s role in CKD-MBD. Additionally, inflammatory mediators in the bone microenvironment amplify local RANKL/RANK signaling, linking systemic and localized immune responses to bone pathology (44).

#### 4.1.2 Roles of sclerostin and FGF-23

Sclerostin and FGF-23, osteocyte-derived regulators, play pivotal roles in CKD-MBD’s bone abnormalities, modulated by inflammatory cues. Sclerostin, an inhibitor of Wnt/β-catenin signaling, suppresses bone formation and is elevated in CKD, contributing to ABD characterized by low turnover (45). In peritoneal dialysis patients, high sclerostin levels correlate with reduced bone formation rates, a finding supported by bone biopsy data. Conversely, sclerostin may exert a protective effect against VC, as its deficiency in mice exacerbates arterial calcification via enhanced Wnt signaling (46). FGF-23, elevated early in CKD to manage phosphate overload, interacts with inflammatory pathways to influence bone mineralization (44). Its overexpression disrupts osteoblast differentiation and matrix mineralization, potentially through mitogen-activated protein kinase (MAPK) signaling inhibition, as seen in uremic rat models treated with C-type natriuretic peptide (47). The interplay between FGF-23 and inflammation, including IL-6 upregulation, further complicates bone homeostasis in CKD-MBD (44, 48).

### 4.2 Immune-mediated VC

#### 4.2.1 Roles of macrophages

In CKD-MBD, VC is an immune-driven process in which macrophage-mediated chronic inflammation and cytokines play central roles PMID:39867890. The uremic microenvironment disrupts macrophage function, promoting the release of inflammatory factors, which in turn affects mineral metabolism such as calcium and phosphorus disorders and VC (5, 49). Macrophages participate in the process of VC through miRNA regulation such as the miR-125b-5p/TRAF6 (TNF receptor associated factor 6)/NF-κB axis, which is particularly significant in patients with CKD (50). Hyperphosphatemia in CKD activates macrophages, particularly the pro-inflammatory M1 phenotype, to release cytokines like IL-1β, IL-6, and TNF-α, which promote osteogenic differentiation of VSMCs (51, 52). The inflammatory environment is further aggravated by factors specific to CKD. Increased phosphate levels encourage the polarization of M1 macrophages through miRNA and RNA regulatory mechanisms, while senescent macrophages heighten local inflammation and the transdifferentiation of VSMCs via interferon signaling (50, 53). Senescent macrophages exacerbate local inflammation and the transdifferentiation of VSMCs by interfering with interferon signaling that propagates the senescent phenotype. The decline in Klotho expression leads to phosphate retention and exacerbated calcification, and the levels of sKlotho are negatively correlated with the risk of VC (9, 54). The degree of VC is significantly negatively correlated with bone density, reflecting systemic mineral metabolism disorders such as hyperphosphatemia, elevated FGF-23, and abnormal vitamin D levels (55, 56). This phenomenon stems from the abnormal transfer of calcium and phosphorus from the bones to the blood vessels, resulting in pathological bone formation (57). The NLRP3 inflammasome, a key inflammatory platform in macrophages, is activated by uremic toxins such as IS, leading to IL-1β maturation and enhanced calcification (58). Macrophages also contribute directly by releasing matrix vesicles that nucleate calcium phosphate deposits in vessel walls. Recent evidence highlights that sodium-glucose cotransporter 2 (SGLT2) inhibitors, such as canagliflozin, attenuates VC in CKD rats by suppressing NLRP3-mediated cytokine release, suggesting a therapeutic avenue (59). These findings position macrophages as critical mediators linking inflammation to vascular pathology in CKD-MBD.

M2 macrophages play a complex dual role in renal fibrosis and CKD-MBD (60). On one hand, they exhibit anti-inflammatory and tissue repair functions, such as clearing apoptotic debris and secreting IL-10 (61, 62). On the other hand, especially during the late stages of disease or under specific microenvironmental signals (e.g., high phosphate, uremic toxins), their excessive activation or phenotypic instability (imbalance in phenotypic plasticity) can transform them into pathogenic factors (63). At this stage, M2 macrophages directly promote the activation of myofibroblasts and extracellular matrix deposition by secreting factors like TGF-β, thereby exacerbating renal fibrosis. Some of these cells may even participate directly in the fibrosis process through macrophage-myofibroblast transition (MMT) (63). Furthermore, M2 macrophages promote the osteogenic transformation of VSMCs and VC by secreting inflammatory factors (e.g., TNF-α) and pro-calcifying mediators, which aggravates the instability of atherosclerotic plaques, significantly increasing the risk of cardiovascular complications. The heterogeneity of M2 subtypes (e.g., M2a, M2c) further complicates their functions (61, 64). Therefore, a deeper understanding of the mechanisms regulating M2 polarization direction, stabilizing their reparative phenotype, and overcoming their plasticity is crucial for developing strategies that target M2 macrophages to delay renal fibrosis and mitigate cardiovascular risks associated with CKD-MBD.

#### 4.2.2 Osteogenic transformation of VSMCs

Vascular smooth muscle cells undergo an osteogenic transformation in CKD-MBD, driven by immune signals and uremic conditions, culminating in VC. Inflammatory cytokines and uremic toxins, including trimethylamine-N-oxide (TMAO), activate the NLRP3 inflammasome and NF-κB pathways in VSMCs, upregulating osteogenic markers like Runx2 and BMP2 (65). This phenotypic switch is marked by increased expression of alkaline phosphatase and osteocalcin, mirroring bone-forming cells (66). MicroRNA-34a, upregulated by inflammatory stimuli in CKD, further promotes VSMC senescence and calcification by modulating sirtuin 1 and Notch1 signaling. Phosphate excess, a hallmark of CKD, triggers this transformation via MAPK and NF-κB activation, as demonstrated in rat models where zinc supplementation inhibited calcification by enhancing TNFAIP3-mediated NF-κB suppression (67). These pathways highlight the immune-mediated mechanisms driving VSMC osteogenesis in CKD-MBD (59).

Beyond the intrinsic transformation of VSMCs, the vascular microenvironment in CKD-MBD is profoundly shaped by the involvement of diverse immune cells, which synergistically exacerbate calcification and inflammatory responses.

#### 4.2.3 Roles of other immune cells

In CKD-MBD, mast cells drive renal inflammation and fibrosis processes directly by releasing proteases (such as tryptase and chymase) and inflammatory mediators (such as histamine and heparin) (68). Their activated state may serve as a potential biomarker for assessing CKD progression (69). Neutrophil function is significantly disrupted (with impaired chemotaxis, phagocytosis, and reactive oxygen species generation), which not only leads to decreased anti-infection capabilities and a persistent micro-inflammatory state but also may accelerate atherosclerosis by releasing NETs, thereby indirectly promoting cardiovascular complications (70–72). Natural killer (NK) cells are characterized by a reduced number and weakened cytotoxicity in kidney failure, which undermines immune surveillance and exacerbates systemic inflammation and immune imbalance (73, 74). Mast cells are important contributors to the instability of atherosclerotic plaques, potentially mediated by the release of inflammatory factors such as histamine or tryptase (75, 76). In stone formers, the number of mast cells was found to be significantly correlated with cortical calcification, suggesting that mast cell infiltration may locally drive the calcification process (77). The accumulation of uremic toxins and OS can activate innate immune pathways, such as TLR signaling, promoting inflammatory responses. TLR signaling may promote calcification by activating the osteogenic differentiation pathway of VSMCs, with dendritic cells (DCs) being key responsive cells in the TLR pathway (75, 78). Exosomes released by bone marrow mesenchymal stem cells (BMSCs) carry miRNAs that can inhibit VSMC calcification (79). DCs may participate in microenvironmental regulation through the uptake or secretion of exosomes (80). VC is negatively correlated with bone density reduction, a relationship possibly driven by shared inflammatory pathways such as RANKL/OPG imbalance (81, 82). If DCs activate Th17 cells (which secrete IL-17), they may simultaneously exacerbate bone loss and VC (57). Neutrophils release inflammatory factors such as IL-6 and TNF-α, while mast cell degranulation products and dysfunction of NK cells collectively promote a systemic microinflammatory state. This chronic inflammation may influence CKD-MBD through various pathways, such as accelerating VC and interfering with bone metabolism.

Above all, mast cells are central drivers that release mediators such as IL-6 and CXCL10 in response to estrogen deficiency, which impairs bone repair and promotes vascular inflammation (83, 84). DCs function as regulators, modulating bone immunity and the vascular microenvironment through antigen presentation and cytokine secretion (85). NK cells act more as effector cells, amplifying the pathological processes in bone and vasculature during inflammation (86, 87). The interactions among these three cell types within the bone-vascular axis (e.g., mast cell activation of DCs and DCs supporting NK cells) form an immune network; however, the direct integration mechanisms documented in the literature are limited. These mechanisms hold significant importance in diseases such as osteoporosis, atherosclerosis, and post-traumatic bone repair, and targeting these cells (e.g., inhibiting mast cells) may ameliorate the dysregulation of the bone-vascular axis (88, 89).

### 4.3 Impact of uremic toxins on bone and immune cells

Uremic toxins accumulating in CKD profoundly affect bone remodeling and immune function, exacerbating CKD-MBD. IS, a protein-bound toxin, impairs osteoblastogenesis by inhibiting Runx2 via the aryl hydrocarbon receptor (AhR) pathway while promoting osteoclastogenesis through NFATc1 upregulation, disrupting bone homeostasis (90). IS also induces OS and inflammation in immune cells, amplifying systemic inflammation that aggravates bone and vascular damage (91). Similarly, p-cresyl sulfate and advanced glycation end products (AGEs) enhance osteoclast activity and inhibit osteoblast function, contributing to bone demineralization (92). In macrophages, IS triggers toxicity by increasing OS and lipid metabolism abnormalities, linking gut microbiota-derived toxins to atherosclerosis and bone loss (58). Strategies reducing uremic toxin levels, such as dialysis optimization or AhR antagonists like resveratrol, may mitigate these effects, offering potential therapeutic benefits.

## 5 Discussion

The intricate interplay between immune dysregulation and CKD-MBD significantly amplifies clinical risks, notably fracture susceptibility, cardiovascular complications, and mortality. In CKD, chronic inflammation–evidenced by elevated TNF-α–exacerbates bone resorption and compromises bone quality, increasing fracture risk beyond what traditional mineral metabolism markers predict (93). This inflammatory milieu also promotes VC, a key contributor to cardiovascular morbidity, as TNF-α enhances both bone turnover and arterial stiffness (94).

Conventional therapies for CKD-MBD–phosphate binders, vitamin D analogs, and calcimimetics–often fail to address immune-driven pathology (95). For example, denosumab effectively inhibits bone resorption by suppressing RANKL; however, it poses a significant risk of hypocalcemia in patients with CKD-MBD, particularly in those who are dialysis-dependent or have advanced CKD (6, 96–98). This risk arises from the compounded effects of bone resorption inhibition and the inherent calcium-phosphate metabolism disorders associated with CKD, as well as secondary hyperparathyroidism.

Persistent VC despite phosphate control highlights the need for novel strategies targeting inflammation and osteoimmunological pathways (99, 100). Therapeutic strategies targeting immune mechanisms in CKD-MBD are gaining traction, with anti-inflammatory and mineral-regulating approaches showing promise. Cytokine inhibitors and antioxidants, such as those explored in preclinical models, reduce inflammation-driven bone turnover and VC, offering a complementary approach to conventional therapies (100). Cytokine inhibitors such as anti-RANKL and IL-1β targeting pro-inflammatory pathways may improve inflammation and cardiovascular outcomes in CKD (34, 101). Novel strategies involving antioxidants (Nrf2 activators, NOX inhibitors) show greater potential but must balance clinical risks (102–104). The bone protective effects of denosumab are well established; however, its application in advanced CKD requires monitoring of calcium and phosphorus metabolism disorders, including the risk of hypocalcemia (105, 106). Future research should focus on precision anti-inflammatory/antioxidant therapies targeting CKD-MBD, such as combined strategies targeting RANK or the Nrf2 pathway, while strictly evaluating their long-term impacts on mineral metabolism.

Mineral metabolism regulation remains critical, with phosphate binders, vitamin D analogs (e.g., paricalcitol), and calcimimetics effectively lowering PTH and phosphorus levels, yet their impact on inflammation is less consistent (49, 107). Emerging therapies, including anti-sclerostin antibodies and FGF-23 inhibitors, target the osteoimmune axis directly, potentially improving bone integrity while mitigating systemic inflammation (108). For instance, romosozumab, an anti-sclerostin agent, has demonstrated efficacy in hemodialysis patients by enhancing bone mineral density (BMD), though its long-term effects on cardiovascular outcomes remain uncharted (109). Vitamin D receptor activators (VDRAs) directly influence the immune-inflammatory aspects of CKD-MBD by regulating the NF-κB pathway, T cell differentiation, and the expression of pro-inflammatory factors (110–112). This regulation has the potential to improve patients’ immune deficiencies and inflammatory status. SGLT2 inhibitors have led to significant advancements in the treatment of CKD; however, their clinical translation is hindered by cognitive biases, subgroup heterogeneity, and risks associated with bone metabolism. The safety and applicability of anti-sclerostin antibodies in CKD necessitate further research for validation. Future efforts should focus on promoting personalized medication strategies and enhancing clinical safety monitoring (113–115). SGLT2 inhibitors have been confirmed in clinical studies to improve biochemical indicators related to CKD-MBD, and their cardioprotective and nephroprotective effects are clear (114, 116). However, the issue of insufficient evidence in non-diabetic CKD populations needs to be addressed. Preclinical studies on anti-bone sclerosis protein antibodies have shown potential for regulating bone metabolism, but cardiovascular risks have limited clinical translation, necessitating the urgent design of new strategies for safety optimization targeted at the CKD-MBD population (117–119). Existing trials have significant limitations in sample size, follow-up duration, endpoint indicators, and population representativeness. Future research should focus on large-scale, long-term studies targeting heterogeneous populations, particularly emphasizing treatment optimization within the framework of precision medicine. These advances highlight a shift toward personalized interventions, yet their integration into clinical practice awaits robust validation.

Despite these insights, significant limitations hinder the translation of immune-focused CKD-MBD research. The reliance on short-term surrogate endpoints, such as PTH or BMD changes, limits understanding of long-term clinical benefits, particularly regarding fracture prevention and cardiovascular mortality (120). Variability in study designs and patient cohorts–often excluding early-stage CKD or non-dialysis populations–further obscures the generalizability of findings (121). Future research should prioritize longitudinal trials to delineate the causal roles of specific inflammatory pathways (e.g., TNF-α, IL-6) and their therapeutic modulation across all CKD stages. Moreover, developing biomarkers that integrate inflammation, bone turnover, and vascular health could refine risk assessment and treatment monitoring, addressing current gaps in precision medicine for CKD-MBD.

## 6 Conclusion

This review highlights the pivotal role of immune dysregulation in the pathogenesis of CKD-MBD, demonstrating how chronic inflammation and aberrant immune responses contribute to bone loss, VC, and increased cardiovascular risk in CKD patients. Key findings indicate that pro-inflammatory cytokines, such as TNF-α, drive bone resorption and vascular stiffening, establishing a mechanistic link between immune activation and the skeletal and extra-skeletal manifestations of CKD-MBD (5, 122). Furthermore, immune-mediated pathways, including sclerostin inhibition of Wnt signaling and FGF-23 dysregulation, have emerged as critical regulators of mineral metabolism and potential therapeutic targets beyond conventional phosphate management (123, 124). These insights underscore that addressing immune mechanisms is essential for improving CKD-MBD management, offering a paradigm shift from solely mineral-focused strategies to integrated immune-modulatory approaches.

Looking forward, future research should focus on identifying immune-related biomarkers to enhance risk stratification and personalize treatment in CKD-MBD. Recent studies suggest that gut dysbiosis, a driver of inflammation and mineral imbalance, warrants exploration as a novel therapeutic avenue, potentially through microbiome-targeted interventions (5). Additionally, advanced techniques like single-cell RNA sequencing could elucidate patient-specific immune profiles, paving the way for tailored therapies that address individual variability in CKD-MBD progression (125). The complexity introduced by osteoimmunology and osteomicrobiology highlights both the challenges and opportunities in this field, necessitating longitudinal, collaborative studies to validate these approaches and translate them into clinical practice.

In conclusion, integrating immune mechanisms into CKD-MBD management holds transformative potential for reducing morbidity and mortality in CKD patients. However, significant knowledge gaps remain, particularly regarding the translation of immune-based interventions into effective treatments. We call for further research to bridge these gaps, leveraging emerging technologies and interdisciplinary efforts to improve outcomes in this high-risk population.
