# Supplementary material for: Immune mechanisms in chronic kidney disease-mineral and bone disorder: current insights and therapeutic implications
Source: Front Med (Lausanne). 2025 Oct 9;12:1678640. doi: 10.3389/fmed.2025.1678640 (PMC12546197; doi:10.3389/fmed.2025.1678640)
Supplement: Supplementary file 1 [file Data_Sheet_1.PDF]

Table 1.

Cell Type	Role in Bone Pathology	Role in Vascular Calcification	Key Regulatory Mechanisms	References
M1 Macrophages	Promote osteoclastogenesis & activation; Inhibit osteoblast function.	Secrete pro-inflammatory cytokines (TNF-α, IL-1β, IL-6) inducing VSMC osteogenic transdifferentiation; Release ROS	High-salt milieu; Uremic toxins	42,43
M2 Macrophages	(Impaired function) Delayed bone repair	(Impaired function) Attenuated anti-inflammatory / reparative capacity	High phosphate; Uremic toxins	53-56
Neutrophils	Indirect effects via inflammation	Release NETs promoting inflammation & initial calcification; Vascular infiltration	Chemokines; Uremic toxins	62-64
Dendritic Cells	Antigen presentation; T cell activation	Antigen presentation, T cell activation; Potential direct pro-inflammatory role	TLR signaling; Microenvironmental factors	67,70,72
Mast Cells	Degranulation releases tryptase	Release histamine or chymotrypsin → ↑ endothelial permeability → facilitate calcium deposition	Trypsin-like enzyme release; Macrophage accumulation	67,68
NK Cells	Cytotoxicity against osteoblasts; Secrete IFN-γ → inhibit bone formation	Recognize calcified VSMCs as "stressed cells" → release perforin/granzyme → amplify vascular damage	Uremic toxin suppression	65,66

Table 2.

**Immune cells**	**Changes/Role in CKD-MBD**	**Impact on Disease Pathogenesis**	**Key Mechanisms/Mediators**	**References**
Macrophages	Polarization to pro-inflammatory M1 phenotype; dysfunction; increased senescence.	Promotes VC and renal fibrosis; exacerbates systemic inflammation.	M1: Release IL-1β, IL-6, TNF-α, BMPs; activate NLRP3 inflammasome; secrete calcifying matrix vesicles.	5, 49–53, 58–59, 63
			M2: Secrete TGF-β, promoting fibrosis and calcification.	60–64
T cells	Shift to pro-inflammatory phenotypes (e.g., Th17); impaired function of Tregs.	Promotes bone resorption; exacerbates inflammation and tissue injury; may link bone loss and VC.	Th17 cells secrete IL-17; increased RANKL expression; loss of immunosuppressive function.	5, 44, 57, 85
Neutrophils	Significant dysfunction (impaired chemotaxis, phagocytosis, ROS generation).	Contributes to persistent micro-inflammatory state and increased infection risk; NETs release may accelerate atherosclerosis.	Release pro-inflammatory cytokines such as IL-6, TNF-α; form NETs.	70–72
Mast cells	Increased numbers and activated state.	Drives renal inflammation and fibrosis; contributes to atherosclerotic plaque instability; may promote local VC.	Release of proteases (tryptase, chymase), histamine, heparin, and inflammatory mediators.	68–69, 75–77, 83–84
NK cells	Reduced numbers and weakened cytotoxicity.	Undermines immune surveillance; exacerbates systemic inflammation and immune imbalance.	Impaired cytokine secretion and cell-killing ability.	73–74, 86–87, 89
DCs	Activated via innate immune pathways (e.g., TLR signaling).	May exacerbate bone loss and VC by activating Th17 cells; participates in microenvironment regulation.	Antigen presentation; uptake/secretion of exosomes; activation of adaptive immune responses.	75, 78, 80, 85
